# Multimodal optical imaging of the oculofacial region using a solid tissue-simulating facial phantom

**DOI:** 10.1117/1.JBO.29.8.086002

**Published:** 2024-08-01

**Authors:** Lilangi S. Ediriwickrema, Shijun Sung, Kaylyn C. Mattick, Miranda B. An, Claire Malley, Stephanie D. Kirk, Divya Devineni, Jaylen M. Lee, Gordon T. Kennedy, Bernard Choi, Anthony J. Durkin

**Affiliations:** a University of California, Irvine, Beckman Laser Institute and Medical Clinic, Irvine, California, United States; b University of California, Irvine, Department of Ophthalmology, Gavin Herbert Eye Institute, Irvine, California, United States; c University of California, Irvine, Biostatistics, Epidemiology, and Research Design Unit, Irvine, California, United States; d University of California, Irvine, Department of Biomedical Engineering, Irvine, California, United States; e University of California, Irvine, Department of Surgery, Irvine, California, United States

**Keywords:** oculofacial, periocular, orbital, spatial frequency domain imaging, optical properties, tissue simulating phantom

## Abstract

**Significance:**

Spatial frequency domain imaging (SFDI) applies patterned near-infrared illumination to quantify the optical properties of subsurface tissue. The periocular region is unique due to its complex ocular adnexal anatomy. Although SFDI has been successfully applied to relatively flat *in vivo* tissues, regions that have significant height variations and curvature may result in optical property inaccuracies.

**Aim:**

We characterize the geometric impact of the periocular region on SFDI imaging reliability.

**Approach:**

SFDI was employed to measure the reduced scattering coefficient (${\mu_{s}}^{\prime }$) and absorption coefficient ($\mu_{a}$) of the periocular region in a cast facial tissue-simulating phantom by capturing images along regions of interest (ROIs): inferior temporal quadrant (ITQ), inferior nasal quadrant (INQ), superior temporal quadrant (STQ), central eyelid margin (CEM), rostral lateral nasal bridge (RLNB), and forehead (FH). The phantom was placed on a chin rest and imaged nine times from an “en face” or “side profile” position, and the flat back of the phantom was measured 15 times.

**Results:**

The measured $\mu_{a}$ and ${\mu_{s}}^{\prime }$ of a cast facial phantom are accurate when comparing the ITQ, INQ, STQ, and FH to its flat posterior surface. Paired t tests of ITQ, INQ, STQ, and FH $\mu_{a}$ and ${\mu_{s}}^{\prime }$ concluded that there is not enough evidence to suggest that imaging orientation impacted the measurement accuracy. Regions of extreme topographical variation, i.e., CEM and RLNB, did exhibit differences in measured optical properties.

**Conclusions:**

We are the first to evaluate the geometric implications of wide-field imaging along the periocular region using a solid tissue-simulating facial phantom. Results suggest that the ITQ, INQ, STQ, and FH of a generalized face have minimal impact on the SFDI measurement accuracy. Areas with heightened topographic variation exhibit measurement variability. Device and facial positioning do not appear to bias measurements. These findings confirm the need to carefully select ROIs when measuring optical properties along the periocular region.

## 1 Introduction

Periocular inflammatory and infiltrative conditions are of particular importance given the proximity to the eye, thereby raising the risk for dry eyes, exposure keratopathy, eyelid malposition, blurry vision, eye pain, vision loss, double vision, and rarely, extension to the central nervous system. Diseases that affect the region include thyroid eye disease, rosacea, and eczema, as well as basal cell carcinoma, squamous cell carcinoma, melanoma, and vascular malformations. Clinical diagnosis and management of these diseases are oftentimes dependent on invasive, time-intensive, and costly procedures and testing, as well as immunotherapy, surgical resection, and periocular reconstruction. Non-invasive objective approaches to measuring local inflammation and infiltration are therefore needed to better quantify disease activity, progression, and treatment response.

**Fig. 1:**
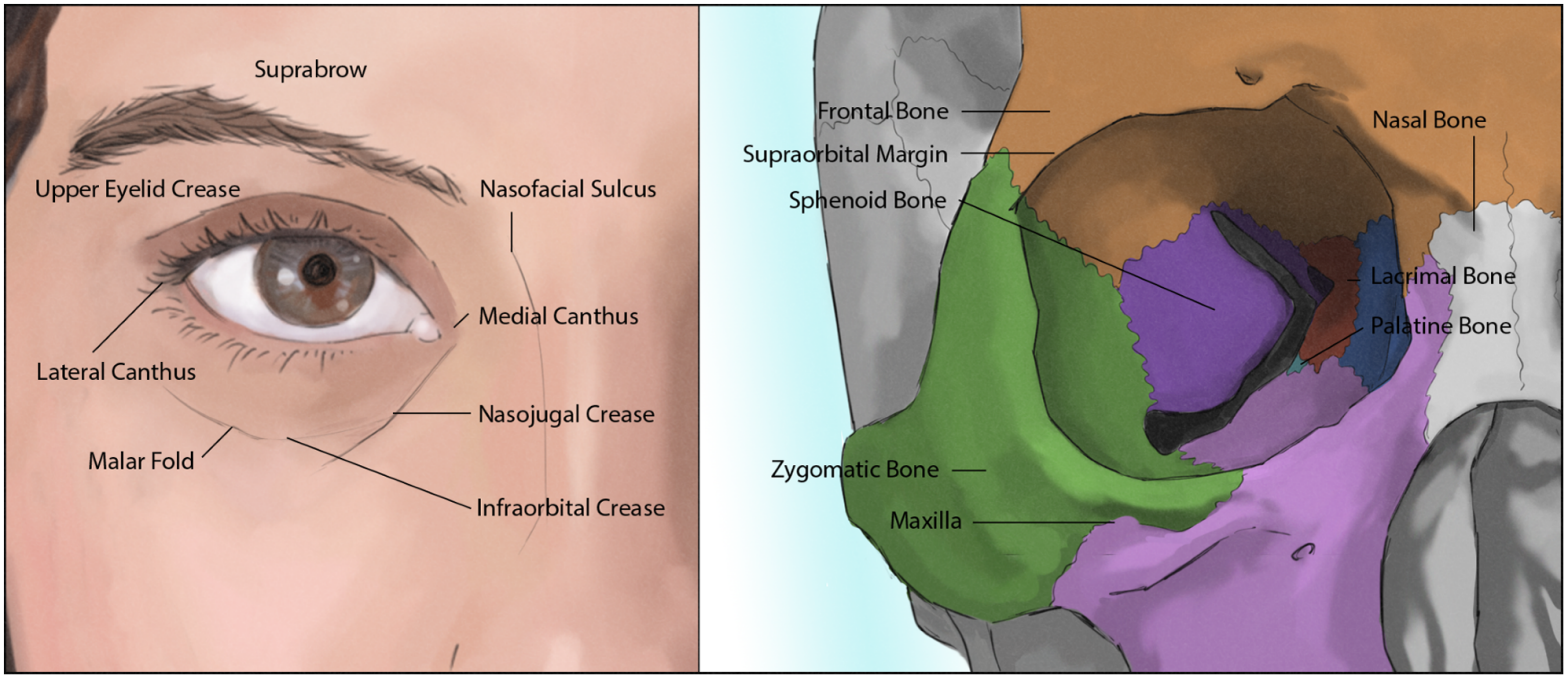
Periocular anatomy. Demarcation of eyelid and canthal structures, in addition to cutaneous folds, creases, and underlying bony framework.

The periocular region is unique due to a complex ocular adnexal anatomy (Fig. 1).^1^ It is comprised of the eyes, including the upper and lower eyelids, eyelashes, and medial and canthal regions, as well as the nose, brow, and midface. The underlying bony framework creates a unique geometric structure with variable height differences, in part affected by the deep orbital bones and structures, in addition to the supraorbital ridge and frontal bone rostrally, nasal bone and malar process of the maxilla medially and inferiorly and zygoma laterally. The soft tissue landmarks are the suprabrow rostrally, medial canthus and nasofacial sulcus medially, nasojugal crease and infraorbital crease inferiorly, and lateral canthus laterally.

Several imaging techniques are available to image the skin, but these probe a superficial depth (Table 1). For example, reflectance confocal microscopy (RCM) is an optical imaging technique that utilizes a single near-infrared (NIR) wavelength (830 nm) to scan the superficial dermis to a penetration depth of ∼200 to 300  μm.^2^ RCM is limited by its use of a single wavelength and interrogates only up to the papillary dermis or upper reticular dermis. Similarly, multiphoton microscopy can provide cellular-level resolution for skin imaging, but it only penetrates up to the superficial dermis, has a limited field of view, and requires eye-safety protocols when used around the periocular region.^3^ Ultrasound, particularly at high frequencies, achieves a resolution of subcutaneous lesions comparable to measurements obtained via histology, but it is also limited by a penetration depth of ∼1.5  mm at 100 Hz.^4^ Optical coherence tomography (OCT) uses an infrared laser to image the skin with ∼2  mm of penetration depth. Although a large meta-analysis found some evidence for the role of OCT in identifying basal cell carcinoma (BCC) when compared with visual inspection and dermoscopy, no evidence-based guidelines currently exist for using OCT to diagnose or monitor cutaneous inflammatory disease and non-BCC malignancies.^5^^,^^6^ OCT possesses advantages in terms of fast acquisition, high resolution of skin morphology, and detection of particulate formations, such as vaccine-loaded carriers, nanoshells, and nanoparticles.^7^^–^^9^ It is however limited in acquiring high-resolution images of non-particulate skin structures, similar to that of RCM.^10^

**Table 1:** Subsurface imaging technology comparison.

Technology	Modality	Penetration depth
RCM	Single NIR wavelength	200 to 300 μm
High-frequency ultrasound	Sound	1.5 mm
OCT	Infrared wavelengths	2 mm
SFDI^a^	Visible to NIR wavelengths	2 to 5^ mm

a Assumes semi-infinite medium with homogenous optical properties. ^Upper limit optimized at wavelengths beyond 850 nm.

Spatial frequency domain imaging (SFDI) is a non-invasive, non-contact wide-field imaging platform that applies patterned NIR illumination to quantify the spatially resolved optical properties of *in vivo* subsurface tissue and can be performed in the clinical setting.^11^^–^^19^ It specifically allows for visualization of tissue volumes up to 5 mm beneath the surface. A region of interest (ROI) along a targeted tissue is illuminated over a range of wavelengths, and the illuminations are projected and recorded by a camera to extract the diffuse reflectance at each specific wavelength and frequency. Ultimately, reduced scattering and absorption coefficients are determined by fitting to a known forward model.^20^ Validation studies were performed by our group using tissue-simulating phantoms with a reported accuracy of ∼6% and 3% in absorption and reduced scattering parameters, respectively.^20^ SFDI can quantify tissue function and structure and has successfully predicted cutaneous flap survival during surgical reconstruction, laser treatment response in port-wine stains, and burn severity.^19^^,^^21^

Although SFDI has been successfully applied to *in vivo* tissues that are relatively flat and unvarying in terms of height (e.g., volar forearm, abdomen, dorsum), measurements on portions of the anatomy that have significant height variations and curvature may result in optical property inaccuracies due to limitations of the height and curvature correction algorithms. Correction algorithms for these issues exist and are used; they have limitations, but developmental efforts continue.^22^ To address this gap, we characterize the potential geometric impact of neighboring bony (nasal process of the maxilla, nasal bone, supraorbital rim, lateral zygoma) and soft tissue (nasal cartilage and anterior projection, brow ptosis) regions on the accuracy of SFDI measurements by fabricating, imaging, and analyzing a facial phantom comprised of poly(dimethylsiloxane) (PDMS) embedded with titanium dioxide granules (scatterer) and India ink (broadband absorber), to identify robust and accurate ROIs to ultimately image human patients. These PDMS phantoms are developed in the lab given their portability, ability to mimic *in vivo* biologic conditions, and overall durability.^23^^–^^25^

## 2 Materials and Methodology

### 2.1 Facial Phantom Fabrication

The facial phantom was fabricated using the previously established methodology.^24^ The base phantom material was PDMS (P4, Reynolds Advanced Materials, Chicago, Illinois, United States). Scattering was provided by adding titanium oxide (${\mathrm{TiO}}_{2}$) powder [titanium (IV) oxide, anatase 248576, Sigma Aldrich, St. Louis, Missouri, United States], and the NIR absorption was obtained using India Ink (Black India Ink, Higgins, Massachusetts, United States). First, a negative cast of a facial structure was created using an alginate-based casting material (LifeMold Alginate Molding Powder, EnvironMolds, Summit, New Jersey, United States), and a facial mold was created from a realistic silicone rendering of a face (FXmasters, Kyiv, Ukraine).

The PDMS phantom was prepared by a three-step process. First, ${\mathrm{TiO}}_{2}$ powder (1 g/kg) was added to the PDMS curing agent (“component A”), and the mixture was sonicated for 3 h, with regular mixing to break up clumps and create a homogenous mixture. India Ink (∼0.26  g/l) was added to the base silicone (“component B”) and mixed using a mixing propeller attached to an electric drill for ∼3  min until the mixture became grossly homogenous in color and texture. Previous studies have indicated that this process yields PDMS phantoms having a reduced scattering coefficient of $∼1\;{\mathrm{mm}}^{-1}$ and an absorption coefficient of $∼0.03\;{\mathrm{mm}}^{-1}$ at a wavelength of 700 nm.^24^ Finally, the two components were combined and mixed using the mixing propeller with a drill for ∼5  min and poured into the negative cast.

The cast and the phantom were placed in a vacuum chamber and degassed using a rotary vacuum pump at a pressure of 30 to 60 mbar for approximately an hour. After that, they were placed atop a level surface and left to cure for 72 h. The facial phantom was then removed from the mold and set aside for 7 days to fully cure before characterization.

### 2.2 Imaging

SFDI measurements were obtained using the Reflect RS™ (Modulim, Inc., Irvine, California, United States) to collect calibrated diffuse reflectance images over a $15\times 20\;{\mathrm{cm}}^{2}$ field of view. The SFDI light sources consist of eight light-emitting diode sources with eight center wavelengths (470 to 851 nm). For each wavelength, sinusoidal light patterns at five spatial frequencies (0, 0.05, 0.10, 0.15, and 0.2/mm) were projected onto the tissue, following conventional procedures previously used.^26^ The Modulim Reflect RS device has built-in cross polarizers, which minimize glare (i.e., surface specular reflection).

**Fig. 2:**
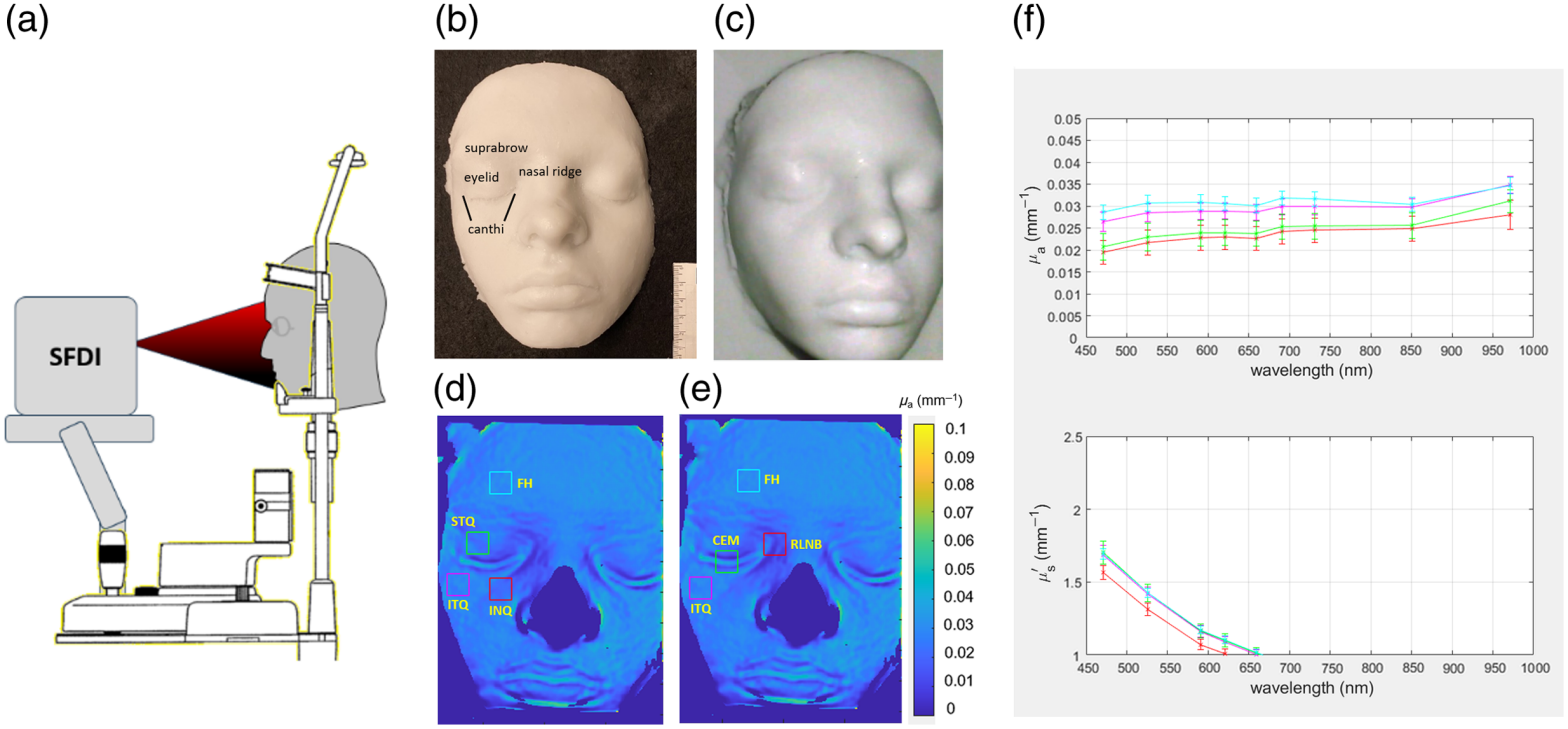
(a) SFDI system consisting of a light source projecting spatially modulated light onto a facial phantom positioned in a chin rest. Facial phantom with delineated periocular anatomy captured (b) “en face” and (c) “side profile” with (d, e) identified ROI, ITQ, INQ, STQ, FH, RLNB, and CEM used to measure ${\mu_{s}}^{\prime }$ and $\mu_{a}$ coefficients. (f) Reduced scattering plots for each measured optical property.

The facial phantom was affixed to a custom-made cart-based chin rest with dual forehead (FH) apposition to simulate human patient positioning and was imaged in a dark room to minimize the effects of ambient illumination [Figs. 2(a) and 2(b)]. The phantom was imaged via an “en face” and “side profile” (nasal tip over contralateral cheek junction) at each of the nine image acquisitions. The posterior flat surface of the phantom was then imaged 15 times by flipping the phantom and re-affixing the phantom to the cart-based chin rest with dual FH apposition. Each region was imaged three consecutive times using the device. A single acquisition, which includes a single image taken at each of the eight wavelengths at all five spatial frequencies, took ∼30  s to complete. Using the software accompanying the instrument (Modulim Inc.), data processing of three repetitions for all anatomical locations on a single-facial phantom took less than 10 min. Following the conventional SFDI measurement technique, a flat PDMS-based tissue-simulating reference phantom with known optical properties was measured at each imaging time point under the same lighting conditions as that of the facial phantom. These imaging data were used to calibrate the SFDI data.

### 2.3 SFDI Data Extraction and Analyses

Raw images obtained from the backscattered light from the facial phantoms were calibrated with images from the reference standard and processed using the “MI-Analyze” software suite (Modulim, Inc.). The processing utilizes a well-established computational Monte Carlo light transport forward model to obtain the reduced scattering coefficient (${\mu_{s}}^{\prime }$) and absorption coefficient ($\mu_{a}$) at each wavelength.^20^ The model generates a 768×768 element look-up-table with coefficient ranges of $0\leq \mu_{a}\leq 3.0$ (${\mathrm{mm}}^{-1}$) and $0.01\leq {\mu_{s}}^{\prime }\leq 4.0$ (${\mathrm{mm}}^{-1}$), with anisotropy and refractive index values fixed at 0.8 and 1.4, respectively.^26^ The algorithm used to process the acquired data specifically used multiple phantom calibrations to correct for intensity changes due to vertical translation in height and variations in surface angles, which accounts for the largest source of error in reflectance in live subjects.^22^

ROIs include the inferior temporal quadrant (ITQ), inferior nasal quadrant (INQ), superior temporal quadrant (STQ), central eyelid margin (CEM), rostral lateral nasal bridge (RLNB), and FH (control); these were selected by a single individual to minimize variability [Figs. 2(c) and 2(d)]. The ITQ was measured ∼1  cm below the lateral canthus, the INQ was measured 1 cm below and temporal to the medial canthus, the STQ was measured in the region between the lateral upper eyelid and brow, and the FH was measured 1 cm above the brow on the same side as the imaged periocular region. The CEM was measured along the central area overlying the eyelid margins, and the RLNB was measured ∼1  cm rostral and medial from the medial canthal region. ROIs were arbitrarily chosen from the center of the posterior flat surface of the facial phantom as points of comparison.

### 2.4 Phantom Surface Characterization

The facial phantom was scanned digitally employing the Creality Lizard 3D scanner (Shenzhen, China) to obtain surface curvature measurements. Subsequently, the scanned data was transferred to Autodesk Fusion 360 (San Francisco, California, United States), by which each chosen ROI was delineated and vertically measured utilizing the inspect function. The corresponding curvature classification, denoted as either convex or concave, was recorded.

### 2.5 Statistical Analyses

SFDI measures of ${\mu_{s}}^{\prime }$ and $\mu_{a}$ were computed for each ROI to generate 12 measured values (six pairs of absorption and scattering values) per facial phantom laterality. Comparisons between the angle profiles (“en-face” versus “side profile”) from the same ROI underwent evaluation through the application of a two-sample t test. Meanwhile, a paired t test specifically quantified the within-face comparisons between the ROIs on $\mu_{a}$ and ${\mu_{s}}^{\prime }$. Confidence intervals are presented on these differences at the 99.9% level to account for multiple comparisons.

## 3 Results

### 3.1 Precision and Accuracy in Facial Phantom Measurement

**Fig. 3:**
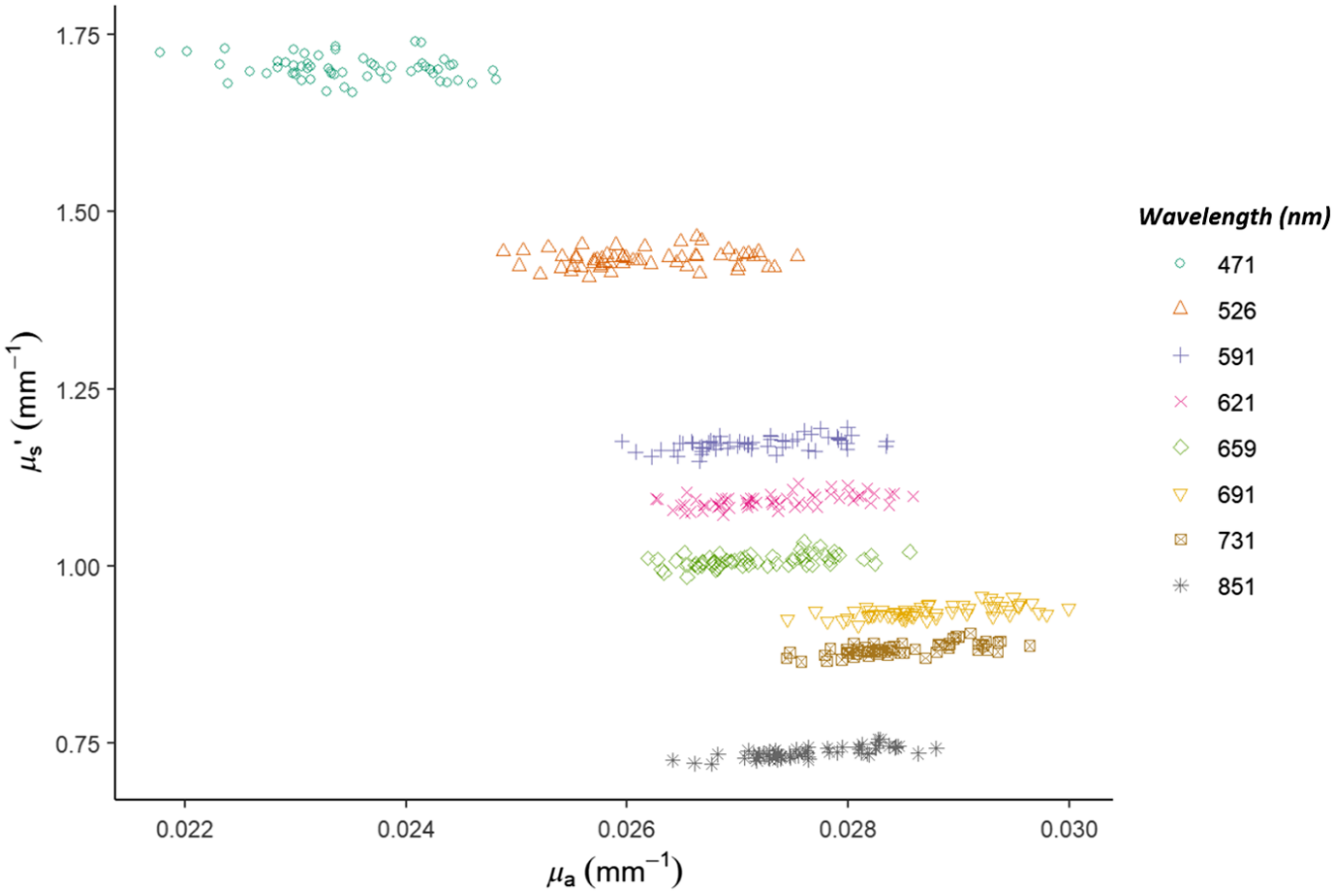
Optical properties of the posterior flat surface of the facial phantom at all wavelengths.

The mean absorption ($\mu_{a}$) and reduced scattering (${\mu_{s}}^{\prime }$) coefficients for the back surface of the phantom were 0.0275 and $0.736\;{\mathrm{mm}}^{-1}$, respectively, and the standard deviations were 0.000577 and $0.00685\;{\mathrm{mm}}^{-1}$ at the 851 nm wavelength (Table 2). The measured values for the absorption and reduced scattering coefficients reveal tight clustering of the data across each wavelength (Fig. 3).

**Table 2:** Optical properties of the posterior flat surface of the facial phantom at 851 nm.

$\mu_{a}$ (${\mathrm{mm}}^{-1}$)		${\mu_{s}}^{\prime }$ (${\mathrm{mm}}^{-1}$)	
Mean	Standard deviation	Mean	Standard deviation
0.0275	0.000577	0.736	0.00685

**Fig. 4:**
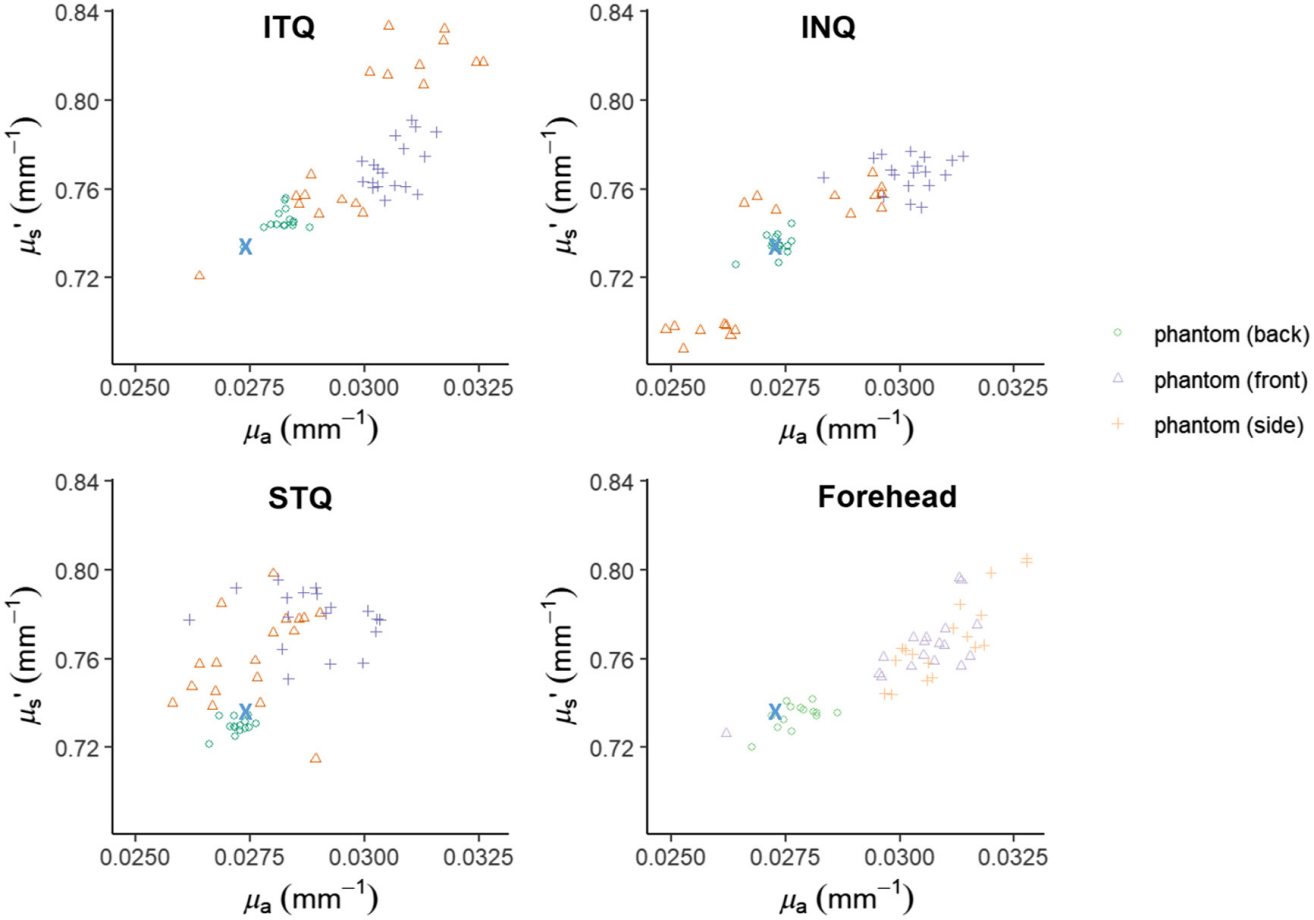
Facial phantom optical property scatter plot. The distribution of measurements for the back of the phantom, “side profile,” and “en face” for ROIs at 851 nm.

These values were precise for the back surface of the phantom, as well as for each of the periocular ROIs (INQ, ITQ, STQ) and FH of the facial phantom as repeated measurements of each area resulted in similar values with coefficients of variation for $\mu_{a}$ at 0.054, 0.065, 0.036, and 0.041 (${\mathrm{mm}}^{-1}$) and for ${\mu_{s}}^{\prime }$ at 0.047, 0.043, 0.028, and 0.02 (${\mathrm{mm}}^{-1}$), respectively (Fig. 4).

These values were also accurate in that the periocular ROIs and FH of the facial phantom were similar in value to that of the back surface of the phantom, with a maximum mean difference of 0.003 to 0.031 ${\mathrm{mm}}^{-1}$ for $\;\mu_{a}$ and ${\mu_{s}}^{\prime }$ respectively, which are not significant (Table 3). The optical properties for each ROI at all eight measured wavelengths (470 to 851 nm) are also provided (Table S1 in the Supplementary Material).

**Table 3:** Facial phantom ROI optical properties at 851 nm. Two sample t tests with 99.9% confidence intervals are presented.

$\;\mu_{a}$ (${\mathrm{mm}}^{-1}$)					${\mu_{s}}^{\prime }$ (${\mathrm{mm}}^{-1}$)				
ROI	“en face”	Back	Difference	Percent difference	ROI	“en face”	Back	Difference	Percent difference
ITQ	0.03 (0.029, 0.031)	0.028 (0.028, 0.029)	−0.002 (0.001, 0.003)	6.9 (4.2, 9.6)	ITQ	0.785 (0.766, 0.805)	0.746 (0.737, 0.754)	−0.04 (0.023, 0.056)	5.6 (4.1, 7.2)
INQ	0.027 (0.024, 0.03)	0.027 (0.027, 0.028)	0 (−0.003, 0.003)	0 (−10.3, 11.4)	INQ	0.729 (0.704, 0.755)	0.735 (0.728, 0.742)	0.005 (−0.028, 0.017)	−0.7 (−3.7, 2.4)
STQ	0.028 (0.026, 0.029)	0.027 (0.027, 0.028)	0 (−0.001, 0.002)	4.2 (1.6, 7)	STQ	0.761 (0.739, 0.783)	0.73 (0.725, 0.735)	−0.031 (−0.011, 0.051)	4.2 (1.6, 7)
FH	0.03 (0.028, 0.032)	0.028 (0.027, 0.029)	−0.003 (0.001, 0.004)	9.9 (6.5, 13.4)	FH	0.765 (0.735, 0.795)	0.734 (0.726, 0.743)	−0.031 (0.004, 0.057)	4.2 (2.5, 5.9)
CEM	0.021 (0.019, 0.024)	0.028 (0.028, 0.029)	−0.007 (−0.009, −0.005)	−23.2 (−29.8, −15.9)	CEM	0.889 (0.853, 0.919)	0.883 (0.875, 0.892)	0.005 (−0.028, 0.038)	2.5 (−1.5, 6.7)
RLNB	0.02 (0.014, 0.026)	0.029 (0.028, 0.029)	−0.009 (−0.015, −0.003)	−29.5 (−47.8, −4.6)	RLNB	0.747 (0.638, 0.857)	0.88 (0.867, 0.892)	−0.132 (−0.239, −0.025)	−12.2 (−23.1, 0.1)

**Fig. 5:**
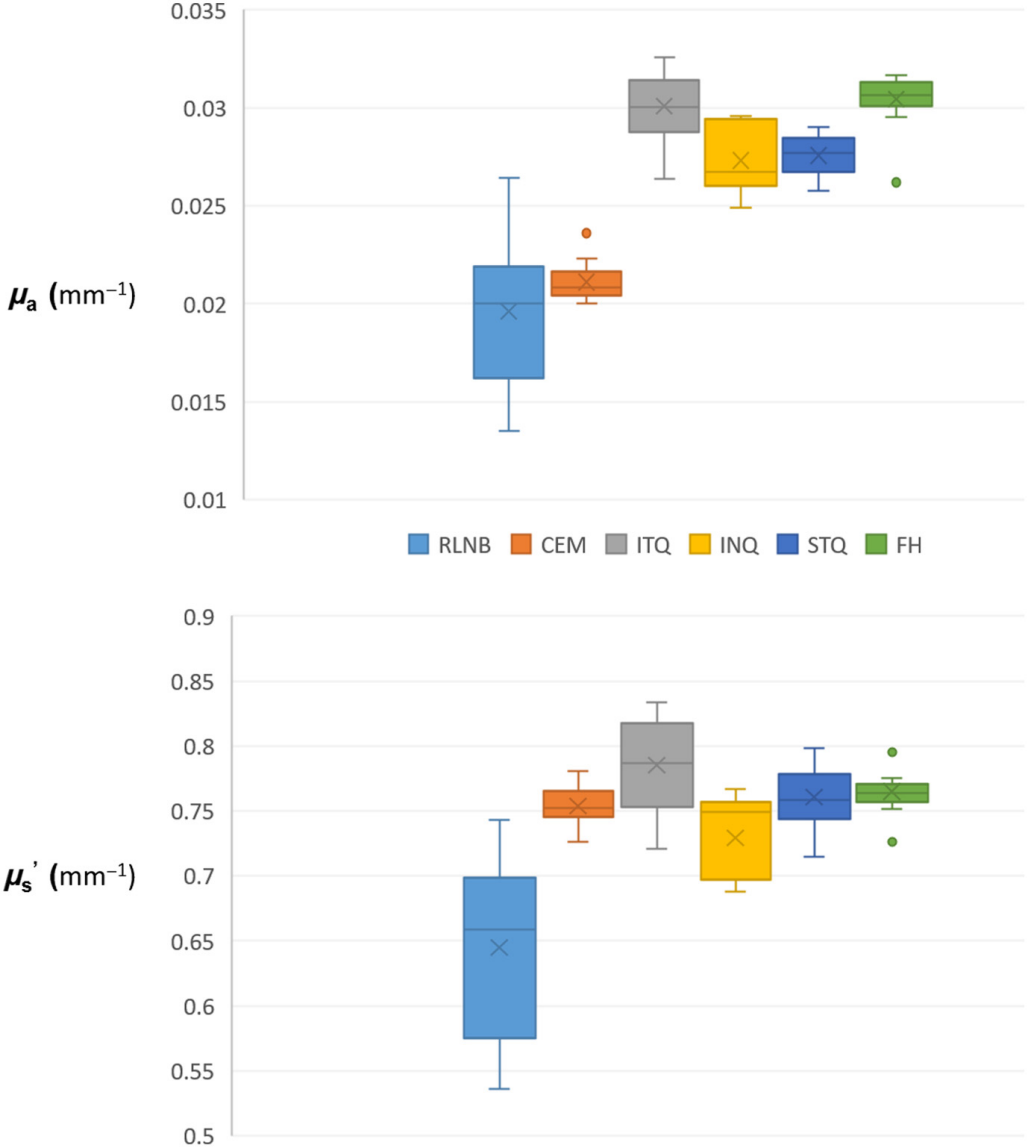
Box and whisker plots demonstrating the artifactual decrease in $\mu_{a}$ for both the RLNB and CEM regions and the artifactual decrease in ${\mu_{s}}^{\prime }$ for RLNB.

**Fig. 6:**
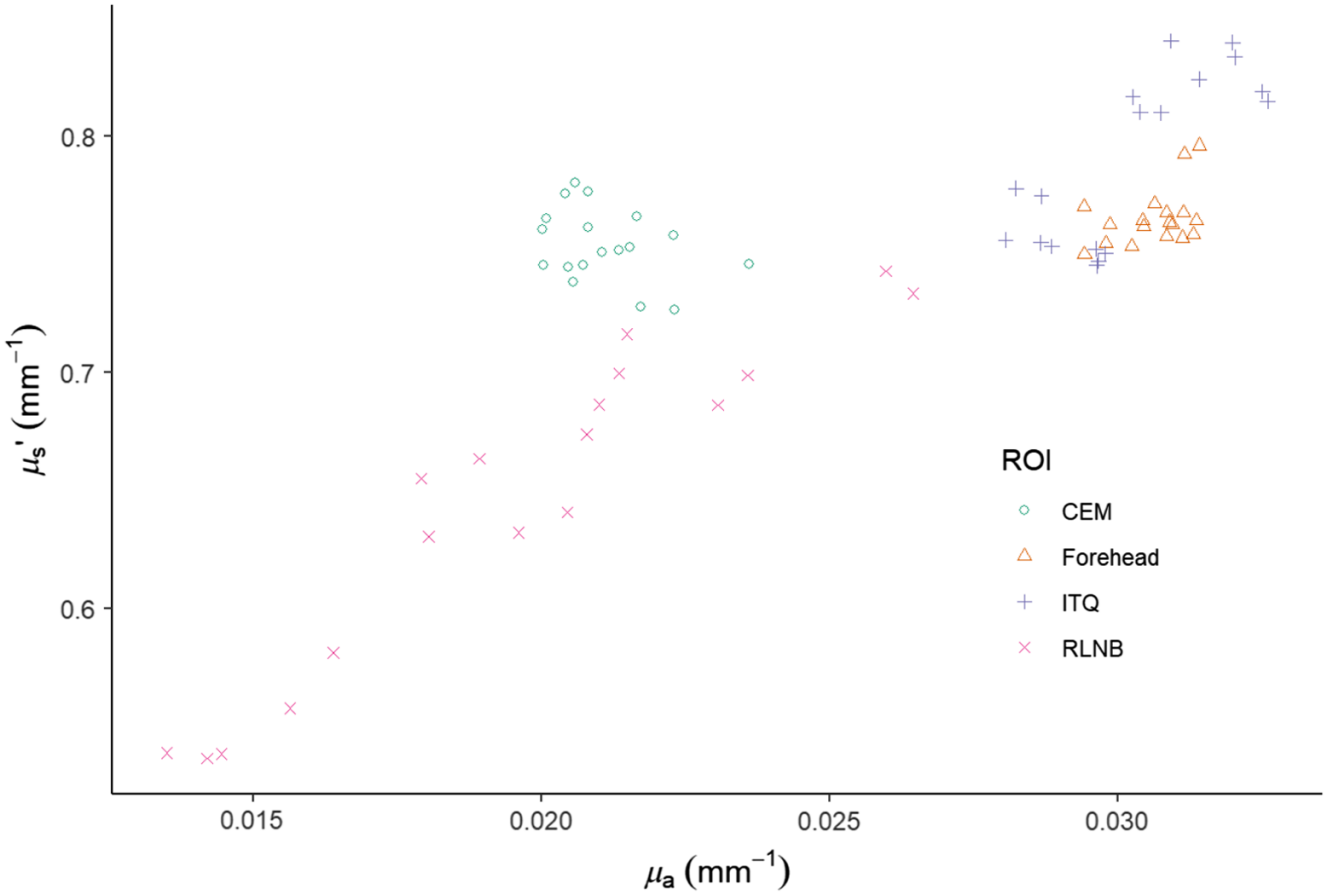
Mean optical property distributions. The RLNB demonstrates artificially decreased values in $\mu_{a}$ ($0.02\;{\mathrm{mm}}^{-1}$ mean) and ${\mu_{s}}^{\prime }$ ($0.645\;{\mathrm{mm}}^{-1}$ mean), with increased standard deviations (0.0038 and $0.068\;{\mathrm{mm}}^{-1}$, respectively), whereas the CEM demonstrates artificially decreased values in $\mu_{a}$ ($0.02\;{\mathrm{mm}}^{-1}$ mean) with a tight fit ($0.001\;{\mathrm{mm}}^{-1}$ σ), compared with the $\mu_{a}$ of the FH ($0.03\;{\mathrm{mm}}^{-1}$ mean, $0.001\;{\mathrm{mm}}^{-1}$ σ) and ITQ ($0.03\;{\mathrm{mm}}^{-1}$ mean, $0.002\;{\mathrm{mm}}^{-1}$ σ) at 851 nm.

However, ${\mu_{s}}^{\prime }$ of the RLNB was artificially decreased at $0.645\;{\mathrm{mm}}^{-1}$ with an increased spread of data [$0.068\;{\mathrm{mm}}^{-1}$ standard deviation (σ)], compared with ITQ ($0.79\;{\mathrm{mm}}^{-1}$ mean, 0.036 σ), INQ ($0.73\;{\mathrm{mm}}^{-1}$ mean, $0.03\;{\mathrm{mm}}^{-1}$ σ), STQ ($0.761\;{\mathrm{mm}}^{-1}$ mean, 0.02 σ), and FH ($0.765\;{\mathrm{mm}}^{-1}$ mean, $0.015\;{\mathrm{mm}}^{-1}$), whereas the CEM ($0.754\;{\mathrm{mm}}^{-1}$ mean, 0.15 σ) was minimally impacted (Figs. 5 and 6).

On the contrary, the $\mu_{a}$ for both CEM ($0.021\;{\mathrm{mm}}^{-1}$ mean, $0.0009\;{\mathrm{mm}}^{-1}$ σ) and RLNB ($0.02\;{\mathrm{mm}}^{-1}$ mean, $0.0038\;{\mathrm{mm}}^{-1}$ σ) was artifactually lower compared with ITQ ($0.03\;{\mathrm{mm}}^{-1}$ mean, $0.002\;{\mathrm{mm}}^{-1}$ σ), INQ ($0.027\;{\mathrm{mm}}^{-1}$ mean, $0.002\;{\mathrm{mm}}^{-1}$ σ), STQ ($0.028\;{\mathrm{mm}}^{-1}$ mean; $0.001\;{\mathrm{mm}}^{-1}$ σ), and FH ($0.03\;{\mathrm{mm}}^{-1}$ mean, $0.001\;{\mathrm{mm}}^{-1}$ σ) with increased distribution of data for RLNB ($0.0038\;{\mathrm{mm}}^{-1}$ σ) (Figs. 5 and 6).

### 3.2 Device Position and Angle of Incidence Do Not Impact Measurements in Specific Regions of the Face

There is inevitable variability in patient positioning within the chinrest (patient positioning error), as well as aligning the light source in front of the patient (technician error). We therefore imaged the facial phantom from both an “en face” and “side profile” vantage point, after attaching it to the chin rest. The largest estimated mean difference in ROI-specific imaging coefficients for ITQ, INQ, STQ, and FH between the “en face” and “side profile” angles of capture is 0.003 and $0.038\;{\mathrm{mm}}^{-1}$ for the $\mu_{a}$ and ${\mu_{s}}^{\prime }$, respectively. Given the scale of the coefficients, these differences were judged to be negligible (Table 4).

**Table 4:** Impact of light source positioning on facial phantom ROI optical properties at 851 nm. Two sample t tests with 99.9% confidence intervals are presented.

$\mu_{a}$					${\mu_{s}}^{\prime }$				
ROI	“en face”	“side profile”	Difference	Percent difference	ROI	“en face”	“side profile”	Difference	Percent difference
ITQ	0.03 (0.029, 0.031)	0.031 (0.03, 0.032)	−0.001 (−0.002, 0.001)	−1.8 (−5.4, 2.1)	ITQ	0.785 (0.766, 0.805)	0.77 (0.757, 0.784)	0.015 (−0.009, 0.039)	1.9 (−0.3, 4.2)
INQ	0.027 (0.024, 0.03)	0.03 (0.029, 0.032)	−0.003 (−0.005, −0.001)	−9.6 (−18.2, −0.1)	INQ	0.729 (0.704, 0.755)	0.767 (0.754, 0.78)	−0.038 (−0.062, −0.013)	−4.9 (−7.7, −2)
STQ	0.028 (0.026, 0.029)	0.029 (0.027, 0.031)	−0.001 (−0.004, 0.002)	−4.5 (−10.3, 1.7)	STQ	0.761 (0.739, 0.783)	0.778 (0.761, 0.795)	−0.017 (−0.05, 0.016)	−2.2 (−4.7, 0.3)
FH	0.03 (0.028, 0.032)	0.031 (0.03, 0.032)	−0.001 (−0.003, 0.002)	−1.9 (−7.6, 4.1)	FH	0.765 (0.735, 0.795)	0.769 (0.741, 0.798)	−0.004 (−0.036, 0.027)	−0.6 (−4.2, 3.2)

### 3.3 Phantom Surface Estimate of Curvature

A digital 3D scanner was employed to create a curvature map of the various ROIs. The FH, ITQ, INQ, and STQ were found to be convex, whereas the RLNB and CEM were concave (Table 5).

**Table 5:** Facial phantom surface curvature determination.

ROI	Curvature
FH	Convex
ITQ	Convex
INQ	Convex
STQ	Convex
RLNB	*Concave*
CEM	*Concave*

Note: Concave surface curvatures are italicized, identifying the ROIs with error prone optical measurements.

## 4 Discussion

By measuring the wavelength-specific absorption and reduced scattering coefficients as a function of spatial frequency, SFDI can quantify tissue optical properties^18^^,^^19^ that will enable differentiation of normal tissue from tissue undergoing pathologic transformation. Its ability to penetrate up to 5 mm helps differentiate it from OCT, ultrasound, and RCM, which can only penetrate up to 2 mm in depth and are further limited in the ability to acquire high-resolution images of non-particulate skin structures.^7^^–^^10^ SFDI has demonstrated utility in evaluating burn severity, tissue transfer flap viability following surgical reconstruction, laser treatment efficacy in patients with port-wine stains, and changes in cerebral blood flow following cardiac arrest.^12^^,^^14^^,^^15^^,^^17^^,^^19^ Ongoing studies have enabled quantification of oxy and deoxyhemoglobin concentration as well as oxygen saturation,^27^ characterized *in vivo* tissue hemodynamics, and identified subsurface vascular changes in patients before they are clinically obvious. SFDI therefore has the potential to quantify tissue inflammation and hemodynamics and define which metrics correlate with disease activity. By longitudinally following changes in tissue optical properties, we expect to develop a quantitative SFDI-derived metric to study the kinetics of periocular disease evolution and its response to therapy.

The periocular region, however, possesses a unique geometric landscape that may complicate SFDI imaging within this anatomic region. Although well-established studies have demonstrated SFDI’s utility for *in vivo* imaging of relatively flat anatomic regions, the notable curvature and height heterogeneity of the face may result in measurement errors of optical properties due to existing algorithm limitations. Optimization of these correction algorithms involves calibrating the light source intensity with light during surface profile acquisition and implementing a Lambertian reflectance model-based correction, but the process has yet to be finalized and is confined by height variations up to 3 cm and tilt angles within ±40  deg. Furthermore, the correction algorithm is dependent upon the phase profilometry data quality.^22^

We therefore outline an approach to characterize the impact of the unique periocular anatomy using a fabricated PDMS facial phantom due to its biologic optical similarity to that of human tissue to explore the absorption and reduced scattering coefficients along the periocular region using SFDI. The optical property measurements of each periocular quadrant, i.e., inferior temporal, inferior nasal, and superior temporal, as well as the FH, were reproducible within each cohort, similar in magnitude to each other, and that of the flat back surface of the facial phantom (i.e., no height variability), whereas the CEM and RLNB yielded artifactual data with notable spread in measured values for the RLNB. The minimal variability noted between the “en face” and posterior surface of the phantom may, in part, be due to scattering potentially being greater along the rougher “en face,” or surface, side.

The errors inherent to the RLNB ROI are likely due to significant height and curvature variations, in addition to inherent scattering properties (areas where light is more likely to be redirected), that the surface correction algorithm cannot overcome via correction due to phase unwrapping abnormalities. The CEM, on the contrary, exhibits curvature variations that may have implications in localized areas of increased scattering along a smaller scale than RLNB, specifically along the region where the upper and lower eyelid margins meet. Further, the surface curvature was found to be concave along the RLNB and CEM, whereas the FH, ITQ, INQ, and STQ were found to be convex using a 3D facial scanner and curvature map software platform. Concave surfaces may predispose to error using SFDI as there is the likelihood of secondary illumination. The Reflect RS™ SFDI analysis software (MI Analyze) employs a profile-based correction algorithm to account for varying tissue height and angle with respect to the imager.^28^ The model is able to account for variations in height within 2 cm from the target location and angles of ∼±30  deg to the plane of illumination. The correction algorithm was validated on tilted phantoms and phantoms having different radii of curvature. However, it does not account for the secondary illumination of the tissue by light reflected from surrounding tissue structures within the illumination field of the device.

Practically, the CEM is a suboptimal ROI when imaging patients. This region of the face, in a human subject measurement context, is a thin tissue layer overlying a fluid-filled globe. The boundary conditions of the computational modeling are considerably different than the real case, and thus, the process for determining the optical properties of this volume of tissue would not be expected to produce high fidelity, accurate optical properties. A potential option moving forward would be to interrogate a multi-material phantom with integrated dynamic flow.

Finally, light source positioning (“en face” versus “side profile”) and/or patient positioning do not appear to impact SFDI measurement along the ITQ, INQ, STQ, and FH, further suggesting that the geometry of the selected ROIs along the periocular region has acceptably little influence on SFDI measurements. Collectively, these results suggest that corresponding measurements of human subjects should yield accurate optical property values.

Overall, our findings suggest that the curvature of each chosen ROI most likely impacts absorption, whereas the height variation impacts scattering. All of these findings bear out at all measured wavelengths, with data depicted for 851 nm. These outcomes emphasize the need to carefully choose the ROI along the periocular profile, given its topography and adjacent structures (e.g., nasal profile or supraorbital rim) to minimally affect biologic SFDI measurements. Specifically, ITQ, INQ, STQ, and FH were similar to that of the back flat surface of the facial phantom, suggesting that these ROI are ideal regions to conduct human SFDI analyses. These results, i.e., the impact of curvature, are likely applicable to other tissue surfaces and are not limited to skin-like textures.

Our approach is not without limitations, however. A single facial phantom for a youthful rendering of a female face was used. We therefore did not study the impact of natural variations that can occur within the face particularly along the superior sulcus or brow or evaluate the potential impact of aging on the underlying ligamentous and bony framework. Furthermore, we only assessed the impact of curvature and height variation and will need to further study the roles of underlying proptosis as well as orbital orientation on either side of the face. Finally, human skin is a complex tissue structure, which necessitates the study of a multi-layer region, which is not accounted for via this technique.

## 5 Conclusions

To our knowledge, we describe the first characterization of SFDI performance along the periocular facial region using a facial phantom with known optical properties. By creating an internal control (the posterior flat surface of the facial phantom), the various ROIs around the eyes and adjacent structures were able to be compared with each other to assess precision, reproducibility, and accuracy. The periocular regions, in particular, ITQ, INQ, STQ, and FH, were found to be regions in which optical properties can be determined with high confidence. We have thus characterized the potential geometric impact of neighboring bony and soft tissue regions on the ability of SFDI measurements to accurately determine the optical properties of a fabricated PDMS facial phantom toward identifying robust and accurate ROIs. Based on these results, we cautiously aim to conduct characterization studies of control patients, with the ultimate goal of using the technology to characterize structural and functional changes around the periocular region to aid disease diagnosis and therapeutic response.

## Supplementary Material



## Data Availability

Datasets and analysis codes used in this paper are available upon email request.
