# Supplementary material for: Multimodal optical imaging of the oculofacial region using a solid tissue-simulating facial phantom
Source: J Biomed Opt. 2024 Aug 1;29(8):086002. doi: 10.1117/1.JBO.29.8.086002 (PMC11293559; doi:10.1117/1.JBO.29.8.086002)
Supplement: Supplementary file 1 [file JBO_029_086002_SD001.pdf]

Supplemental Material

Wavelength (nm)	ROI	Mean μ_a (mm^{-1})	Standard Deviation μ_a (mm^{-1})	Mean μ_s' (mm^{-1})	Standard Deviation μ_s' (mm^{-1})
471	CEM	0.018	0.002	1.714	0.041
	Forehead	0.371	0.376	2.094	0.842
	INQ	0.351	0.315	1.931	0.424
	ITQ	0.295	0.255	1.901	0.455
	RLNB	0.015	0.004	1.382	0.140
	STQ	0.470	0.615	2.104	0.877
526	CEM	0.019	0.001	1.422	0.028
	Forehead	0.226	0.211	1.701	0.404
	INQ	0.218	0.175	1.623	0.305
	ITQ	0.182	0.145	1.564	0.223
	RLNB	0.017	0.004	1.159	0.116
	STQ	0.272	0.277	1.663	0.300
591	CEM	0.020	0.001	1.163	0.024
	Forehead	0.097	0.098	1.428	0.297
	INQ	0.091	0.071	1.373	0.237
	ITQ	0.077	0.058	1.297	0.156
	RLNB	0.018	0.004	0.958	0.100
	STQ	0.114	0.100	1.349	0.200
621	CEM	0.020	0.002	1.097	0.022
	Forehead	0.304	3.500	1.368	0.292
	INQ	0.043	0.037	1.313	0.234
	ITQ	0.040	0.030	1.237	0.157
	RLNB	0.019	0.005	0.911	0.099
	STQ	0.057	0.053	1.258	0.176
659	CEM	0.020	0.001	1.008	0.022
	Forehead	0.040	0.042	1.280	0.276
	INQ	0.033	0.027	1.228	0.223
	ITQ	0.032	0.023	1.155	0.152
	RLNB	0.018	0.004	0.834	0.087
	STQ	0.045	0.040	1.172	0.163
691	CEM	0.021	0.001	0.937	0.021
	Forehead	0.053	0.111	1.231	0.279
	INQ	0.045	0.096	1.181	0.228
	ITQ	0.043	0.092	1.106	0.157
	RLNB	0.020	0.004	0.781	0.083
	STQ	0.058	0.110	1.124	0.161
731	CEM	0.021	0.001	0.889	0.019
	Forehead	0.591	7.795	1.227	0.303
	INQ	0.023	0.015	1.173	0.247
	ITQ	0.023	0.014	1.092	0.176
	RLNB	0.020	0.004	0.747	0.078

	STQ	0.031	0.023	1.109	0.173
851	CEM	0.021	0.001	0.754	0.015
	Forehead	0.022	0.013	1.211	0.392
	INQ	0.020	0.010	1.165	0.321
	ITQ	0.020	0.011	1.055	0.245
	RLNB	0.020	0.004	0.645	0.068
	STQ	0.026	0.016	1.065	0.227

Supplemental Table 1. ROI Optical Properties at Each Wavelength
